# The mediating role of physical education course interest in the relationship between perceived autonomy support, feedback, and school attachment

**DOI:** 10.3389/fpsyg.2025.1675108

**Published:** 2025-10-01

**Authors:** Ahmet Enes Sağın, Mehmet Akif Yücekaya, Sinan Uğraş, Barış Mergan, Cenk Temel, Muhsin Duran, Pedro Duarte-Mendes

**Affiliations:** ^1^Faculty of Sports Sciences, Bartın University, Bartın, Türkiye; ^2^School of Physical Education and Sports, Dicle University, Diyarbakır, Türkiye; ^3^Faculty of Sports Sciences, Çanakkale Onsekiz Mart University, Çanakkale, Türkiye; ^4^Faculty of Sports Sciences, Tokat Gaziosmanpaşa University, Tokat, Türkiye; ^5^Faculty of Sports Sciences, Akdeniz University, Antalya, Türkiye; ^6^Department of Sports and Well-Being, Polytechnic Institute of Castelo Branco, Castelo Branco, Portugal; ^7^Sport Physical Activity and Health Research & Innovation Center, Castelo Branco, Portugal

**Keywords:** autonomy support, teacher feedback, physical education interest, school attachment, mediation, student engagement

## Abstract

**Introduction:**

Students’ sense of school attachment is crucial for their academic success and emotional well-being. Previous studies have shown that autonomy-supportive behaviors and constructive feedback from teachers can positively affect students’ motivation and participation, especially in physical education (PE) classes. However, limited research has examined the mediating role of students’ interest in PE in the relationship between teacher support and school attachment. This study investigates the mediating role of physical education course interest in the relationship between perceived autonomy support and perceived teacher feedback and students’ overall school attachment.

**Methods:**

A total of 560 middle school students in Türkiye participated in this study. The study utilized validated scales to assess perceived autonomy support, teacher feedback, interest in PE, and school attachment. Structural Equation Modeling (SEM) and mediation analyses were conducted using JASP software, with bootstrap methods applied to test indirect effects.

**Results:**

Findings revealed that both perceived autonomy support and teacher feedback positively and significantly predicted school attachment. Interest in PE was found to mediate the relationship between these variables and school attachment. Specifically, perceived autonomy support and feedback increased students’ interest in PE, which in turn enhanced their sense of attachment to school.

**Conclusion:**

The study highlights the crucial role of autonomy-supportive teaching and constructive feedback in strengthening students’ interest in PE and promoting school attachment. Teachers who foster a supportive learning climate can significantly enhance students’ educational engagement and emotional connection to school.

## 1 Introduction

Teachers’ autonomy support in physical education (PE) courses plays a crucial role in enhancing students’ intrinsic motivation and interest by providing them with greater control over their learning processes (Deci and Ryan, 2000; Cheon et al., 2012, 2014; Tilga et al., 2021). This increased sense of autonomy encourages active participation in PE and promotes both academic success and school attachment (Vansteenkiste et al., 2004; Ntoumanis, 2005). Recent studies further confirm these findings by demonstrating that autonomy-supportive teaching practices enhance students’ positive emotional engagement, reduce antisocial behavior, and foster a more adaptive classroom climate (Leisterer and Paschold, 2022; Cheon et al., 2022; Jankauskienė et al., 2022).

Similarly, teacher feedback significantly shapes students’ school experiences. Constructive and supportive feedback not only helps students feel competent and valued but also increases their engagement in PE lessons (Koka and Hagger, 2010). While positive feedback contributes to students’ self-esteem and motivation, negative feedback may lead to school burnout and decreased satisfaction (Hattie and Timperley, 2007; Ilies et al., 2007). In this regard, the feedback style adopted by teachers is instrumental in promoting students’ psychological well-being and educational development, particularly in PE, where both physical and mental aspects of growth are emphasized.

Although previous research has examined the effects of autonomy support and teacher feedback on school attachment (Aelterman et al., 2019; Curran et al., 2013; De Meyer et al., 2016), the mediating role of students’ interest in PE within this relationship has not been sufficiently explored. It is proposed that students’ interest in PE may serve as a mechanism that enhances or attenuates the influence of teacher behaviors on school attachment. In this context, the present study aims to investigate whether interest in PE mediates the relationship between perceived autonomy support, teacher feedback, and school attachment. A greater interest in PE is expected to increase students’ participation, responsiveness to feedback, and emotional attachment to school. Understanding this mediating process will offer valuable insights for educators and practitioners aiming to foster school attachment. Moreover, it will inform teachers’ planning and communication strategies by highlighting the importance of student-centered and autonomy-supportive practices. Ultimately, the findings are anticipated to contribute to the development of more effective educational environments and interventions that promote students’ holistic development.

## 2 Theoretical framework

The core concepts discussed in this study, autonomy support, teacher feedback, physical education course interest, and school attachment, form a whole within the framework of Self-Determination Theory (SDT), yet each corresponds to different psychological processes. Autonomy support, which means the support provided by the teacher to the student, feeds the need for autonomy by increasing the individual’s sense of choice and control. Teacher feedback supports the need for competence by contributing to the student’s sense of self-efficacy and guiding the learning process. Physical education course interest is a variable that reflects the student’s motivation and that expresses participation in and enjoyment of the learning process; in this respect, it plays a mediating role, being influenced by both autonomy support and feedback. School attachment is the outcome variable that expresses the sense of belonging that students establish with the school environment and represents the individual’s educational experience holistically.

### 2.1 Physical education course interest

PE courses are considered an essential source of motivation that enables students to participate in more physical activities and be more committed to school. Interest in physical education courses refers to students’ positive attitudes toward physical education course, their motivation, and their willingness to participate in in-class activities. This interest encourages students to develop positive feelings toward physical activity, actively participate in classes, and improve their physical skills (Lobo and Dimalanta, 2024; Simón-Chico et al., 2023). Students’ interest in PE brings active class participation and better physical health (Lee, 2004). Therefore, interest in PE can be considered an essential factor that increases both the physical and psychological well-being of students (Kliziene et al., 2021). SDT suggests that students’ interest in PE courses is related to their level of intrinsic motivation (Deci and Ryan, 2000). Intrinsic motivation enables students to participate effectively in lessons with pleasure and in line with their wishes. PE course interest positively affects not only students’ attitudes toward physical activities but also their general academic achievement and social relationships (Deci and Ryan, 2000; Standage et al., 2005). Therefore, it is evident that interest in PE can positively affect students’ physical, psychological, and social development. Increasing this interest may lead students to participate more actively in lessons and improve their school experience.

### 2.2 Perceived autonomy support

Teacher feedback refers to the constructive and directive feedback students receive during their learning process. It is a powerful tool that positively affects students’ academic achievement and learning processes (Yang et al., 2021). Informative and positive feedback increases students’ motivation and improves their performance. Students’ perceptions of feedback and their ability to use it play a decisive role in their academic achievement. Therefore, it is critical for teachers to develop and implement effective feedback strategies to increase students’ overall academic achievement (Brown et al., 2016). Positive feedback is known to contribute to students feeling valued and successful, which in turn leads them to participate more in lessons (Mouratidis et al., 2008; Van der Kleij et al., 2019; Wright et al., 2011).

### 2.3 School attachment

School attachment, which refers to the sense of belonging and being a part of the school, significantly affects students’ academic achievement, school experiences, and overall life satisfaction (Korpershoek et al., 2020; Özdemir and Koruklu, 2013). Research has consistently shown that school attachment is directly related to increasing academic achievement, reducing absenteeism rates, and decreasing dropout rates (Upadyaya and Salmela-Aro, 2013; Wang and Fredricks, 2014). School attachment also contributes to students’ social–emotional development and positive school experiences (Fredricks et al., 2004). Motivational processes function as mechanisms leading to academic achievement, and active participation increases intrinsic motivation, resulting in higher academic achievement (Erdoğdu, 2020). Schools can improve students’ academic achievement and school experiences by creating a positive school climate, encouraging motivation for academic achievement, and supporting students’ self-efficacy (Klem and Connell, 2004).

### 2.4 Relationship between perceived autonomy support, physical education course interest, and school attachment

Teachers’ autonomy support in PE lessons significantly affects students’ interest and attendance. Research shows that perceived teacher support has positive effects on student participation, especially in behavioral and affective dimensions (Guo et al., 2023; Leisterer and Paschold, 2022). Autonomy support allows students to have control over their learning process, which increases their intrinsic motivation and desire to learn. For example, Carriedo et al. (2023) concluded that creating an autonomy-supportive environment in PE classes contributes to increasing intrinsic motivation among students, meeting basic psychological needs, and achieving moderate to vigorous physical activity levels (MVPA), thus meeting international standards. Teacher autonomy support in PE classes positively affects students’ interest and participation in PE classes by increasing their intrinsic motivation (Guo et al., 2023). This support strengthens behavioral and emotional involvement, thus reinforcing students’ school attachment (Cheon et al., 2012). Additionally, autonomy-supportive environments positively affect students’ overall school attachment by increasing their participation in physical activities (González-Peño et al., 2021; Standage et al., 2005).

Research shows that environments that support autonomy in PE classes contribute to increased perceived autonomy, efficacy, and intrinsic motivation among students (Chang et al., 2016; Ommundsen and Kvalø, 2007). Zhang et al. (2012) found that teachers’ support for autonomy, competence, and relatedness predicted students’ beliefs about expectations, task values, concentration, and persistence in physical education. Moreover, students’ interest in the lessons directly affects their motivation levels and their performance in the lessons. As students’ interest in PE increases, they are likely to participate more actively in the lesson and perceive the autonomy support they receive from their teachers more positively. This, in turn, can increase their overall school attachment and academic achievement (Reeve et al., 2004). For example, Leyton-Román et al. (2020) concluded that creating an autonomy-supportive environment in PE classes contributes to meeting basic psychological needs, increasing intrinsic motivation and participation in physical activity among students.

Students’ interest in PE may enhance motivation, facilitate active participation, and reinforce school attachment by making educational experiences more positive and increasing the effectiveness of teacher feedback. Studies such as Cheon et al. (2012) and Standage et al. (2005) have examined the effect of teacher support on student motivation and its relationship to learning outcomes. However, in most of these studies, the number of models examining teacher feedback and autonomy support together, especially those testing the mediating role of physical education course interest between these two teacher behaviors, is quite limited. This study aims to fill this gap, and it makes a unique contribution to the field by testing the dual mediating role of PE course interest in the effect of both feedback and autonomy support on school attachment. Accordingly, these hypotheses were put forward in this study:

H_1_: Students’ perceived autonomy support from physical education teachers positively and significantly predicts interest in physical education classes.

H_2_: Perceived autonomy support from physical education teachers positively and significantly predicts school attachment.

H_3_: Interest in physical education courses has a mediating role in the state perceived autonomy support from physical education teachers predicts school attachment.

### 2.5 Relationship between perceived feedback from physical education teachers, physical education course interest, and school attachment

Students’ school attachment is of critical importance in educational processes, and increasing this attachment may contribute to improving student achievement and the overall quality of education. School attachment enables students to be more successful in academic and social life, and it helps them to be happier and more satisfied in the school environment. In the literature, the effects of physical education courses on student motivation and attachment have been investigated (Bailey et al., 2009; Goudas et al., 1994; Standage et al., 2005; Simón-Chico et al., 2023; Guo et al., 2023). These lessons are known to increase students’ participation in physical activity, improve their social skills, and generally reinforce positive attitudes toward school (Benítez-Sillero et al., 2022). It is likely that the feedback provided to students can further reinforce these positive effects.

Teacher feedback can positively affect students’ interest in lessons and their attachment to school in general. Emotional support and constructive feedback that teachers provide to students contribute to their academic achievement, emotional state, and personal development (Aslam et al., 2023; Øen et al., 2024). In particular, constructive feedback seems to increase students’ interest in the lessons and help them actively participate (Vu and Nga, 2023). In PE lessons, teachers’ feedback can increase students’ interest in the lesson and help them participate more motivated. In a study conducted by Rajid et al. (2023), it was concluded that increased interest in physical education courses increased students’ achievement in the course. In addition, this can be expected to enhance students’ overall school satisfaction (Standage et al., 2005).

PE course interest has been chosen as an important variable to better understand the effects of teacher feedback and autonomy support. Students’ interest in PE courses can strengthen their overall school attachment by increasing their motivation and active participation in lessons. This interest in the lessons contributes to students’ more positive perception of feedback, thus raising their academic and social success. It is thought that the interest in the PE course will reinforce the effects of the support provided by teachers and make students’ educational experiences more positive.

In this context, the following hypotheses were put forward:

H_4_: Perceived feedback from physical education teachers positively and significantly predicts interest in physical education courses.

H_5_: Perceived feedback from physical education teachers positively and significantly predicts school attachment.

H_6_: Interest in physical education courses has a mediating role in the state that perceived feedback from physical education teachers predicts school attachment.

## 3 Materials and methods

### 3.1 Participants

The research group consisted of 560 students (289 girls, 271 boys) studying in public schools in the Southeastern Anatolia Region of Türkiye. The sample was drawn from five different secondary schools. The inclusion criteria required that students be enrolled in middle school (from 5th grade to 8th grade), attend physical education (PE) classes regularly, possess the cognitive capacity to read and understand the questionnaire, and voluntarily participate with written parental consent.

The distribution by grade level was as follows: 194 students were in 5th grade (34.64%), 165 in 6th grade (29.46%), 110 in 7th grade (19.64%), and 91 in 8th grade (16.25%). In terms of age, the participants ranged between 11 and 15 years: 107 students are 11 years, 164 students are 12 years, 137 students are 13 years, 101 students are 14 years, and 51 students are 15 years.

### 3.2 Data collection tools

The Perceived Teacher Feedback Scale: The scale, developed and adopted again by Koka and Hein (2003) to measure the feedback students perceive from the physical education teacher, was adapted into Turkish by Kara et al. (2018). The perceived teacher feedback scale consists of 14 items, including “positive nonverbal feedback,” “negative nonverbal feedback,” “positive general feedback,” and “performance information.” The scale has a 5-point Likert structure. Confirmatory factor analysis was conducted to test the structure of the scale within this study. It was determined that χ^2^ = 396.345/df = 73, *p* = 0.001, CFI = 0.926, TLI = 0.907, GFI = 0.968, SRMR = 0.044, and RMSEA = 0.089 values were within acceptable limits (Kline, 2016), and construct validity was tested. The reliability values of the data collection tool were determined as *α* = 0.907 and *ω* = 0.868.

The Perceived Autonomy Support from Physical Education Teacher Scale: It was developed by Hagger and Chatzisarantis (2007) for exercise environments to evaluate the autonomy support that the individual perceives from important people to the individual (physical education and sports teacher, coach, and peer). The original name of the scale is “The Perceived Autonomy Support Scale for Exercise Settings-PASSES.” The adaptation of the scale into Turkish was made by Müftüler and İnce (2012) on 589 university students. Burucu (2019) adapted the scale by replacing the expression “free time” in the scale items with “physical education course” and the expression “instructor” with “physical education teacher” in her thesis. The scale has 12 items and a 7-point Likert structure. CFA analysis was conducted to test the structure of the scale for this study. It was determined that χ^2^ = 287.133/df = 52, *p* = 0.001, CFI = 0.958, TLI = 0.947, GFI = 0.967, SRMR = 0.032 and RMSEA = 0.007 values were within acceptable limits. The internal reliability coefficients of the scale were detected as *α* = 0.954 and *ω* = 0.946.

The Physical Education Course Interest Scale: The scale developed by Uğraş and Temel (2020) to determine the interest levels of middle school students in physical education courses has 10 items, one dimension, and is a 5-point Likert scale. It was found that the results of the CFA analysis conducted to test the construct validity of the interest in physical education course scale for this study χ^2^ = 124.128/df = 24, *p* = 0.001, CFI = 0.977, TLI = 0.965, GFI = 0.991, SRMR = 0.026 and RMSEA = 0.086 were within acceptable limits. The internal reliability coefficients of the physical education course interest scale were found to be α = 0.947 and ω = 0.933.

The School Attachment Scale for Children and Adolescents: The scale developed by Hill and Werner (2006) to determine the school attachment levels of children and adolescents consists of 3 dimensions: attachment to school, attachment to teacher, and attachment to friend. The scale has a 5-point Likert structure. Savi (2011) adapted the scale to Turkish culture. In line with the hypotheses formed in this study, the 4-item school attachment dimension was included. According to the CFA results for the construct validity of the scale in this study, χ^2^ = 1.1031/df = 1, *p* = 0.001, CFI = 1, TLI = 1, GFI = 0.996, SRMR = 0.027, RMSEA = 0.176. The internal reliability coefficients of the school attachment scale were α = 0.869 and ω = 0.900.

## 4 Statistical analysis

First, the data were transferred to the JASP 0.16.4 statistical program. Then, the measurement model was tested for the reliability of the construct. While testing the measurement model, factor loadings, Cronbach’s alpha, McDonald’s, χ^2^/df = 1, CFI, TLI, GFI, SRMR, and RMSEA values were examined. After testing the measurement model, the mean, standard deviation, skewness, and kurtosis values were analysed. For the normality analysis of the data, the skewness value between ±3 and the kurtosis value between ±10 was taken as a reference (Kline, 2016). Pearson correlation analysis was performed to determine the relationships between perceived autonomy support, perceived feedback, physical education course interest, and school attachment. For the analysis of the model created according to the hypotheses, mediation analysis was performed in the SEM mediation section of the JASP program. Bootstrap analysis was applied to evaluate the significance of direct and indirect effects between variables in the model tested within the scope of the research (Preacher and Hayes, 2008). In the bootstrap analysis, 5,000 samples were preferred. For the direct and indirect effects between the variables in the model to be considered significant, the criterion of no zero between the lower and upper values in the 95% confidence interval was taken as a basis (Preacher and Hayes, 2008).

## 5 Results

The measurement model provides a reliable and valid representation of the analyzed variables in mediation models. Kline (2016) stated that an ill-defined measurement model would weaken the integrity of the structural model and may lead to incorrect results. Since it is impossible to accurately measure abstract concepts with concrete indicators without a measurement model (Bollen, 1989) and the reliability of constructs increases with a measurement model (Bagozzi and Yi, 1988), the measurement model was tested. Hair et al. (2014). It has been stated that a factor loading value of 0.30 can be considered significant if the sample size is 350 or more. When the measurement model was tested, it was decided that this item should be removed since the factor loading of PF’s 14th item was found to be 0.216 in the factor loadings of PF. The factor loadings of the PECI constructs ranged between 0.794 and 1.076, the factor loadings of the PAS construct ranged between 1.375 and 1.746, the factor loadings of the PF construct ranged between 0.367 and 1.100, and the factor loadings of the SA construct ranged between 0.960 and 1.171. The measurement model χ^2^ = 1983.305/df = 691, *p* = 0.001, CFI = 0.927, TLI = 0.922, GFI = 0.930, SRMR = 0.047, and RMSEA = 0.058 values were within acceptable limits (Kline, 2016), and it was detected that the construct validity was tested. Cronbach’s Alpha values were found to be high for the constructs of Interest (0.952), Perceived Autonomy Support (PAS; 0.954), Perceived Feedback (PF; 0.914), and School Attachment (SA; 0.900), indicating that the internal consistency of the constructs was strong. (Table 1).

**Table 1:** Measurement model.

Factors	Indicator	Estimate	Std. error	z-value	*p*	95% confidence interval		Cronbach’s alpha (*α*)
						Lower	Upper	
Perceived Autonomy Support	PAS1	1.375	0.075	18.312	< 0.001	1.228	1.522	
	PAS2	1.465	0.069	21.257	< 0.001	1.330	1.601	
	PAS3	1.550	0.069	22.569	< 0.001	1.415	1.684	
	PAS4	1.695	0.075	22.501	< 0.001	1.547	1.843	
	PAS5	1.746	0.072	24.409	< 0.001	1.606	1.887	
	PAS6	1.695	0.071	23.967	< 0.001	1.557	1.834	0.954
	PAS7	1.581	0.068	23.167	< 0.001	1.447	1.715	
	PAS8	1.585	0.071	22.439	< 0.001	1.447	1.724	
	PAS9	1.638	0.071	23.073	< 0.001	1.499	1.777	
	PAS10	1.641	0.070	23.391	< 0.001	1.503	1.778	
	PAS11	1.536	0.070	21.984	< 0.001	1.399	1.673	
	PAS12	1.488	0.069	21.576	< 0.001	1.353	1.623	
Perceived Feedback	PF1	0.923	0.046	20.168	< 0.001	0.833	1.013	
	PF2	0.836	0.045	18.579	< 0.001	0.747	0.924	
	PF3	0.981	0.048	20.245	< 0.001	0.886	1.076	
	PF4	0.367	0.061	6.023	< 0.001	0.248	0.486	
	PF5	0.862	0.048	17.983	< 0.001	0.768	0.956	
	PF6	1.055	0.046	22.974	< 0.001	0.965	1.145	
	PF7	1.100	0.050	21.991	< 0.001	1.002	1.198	0.914
	PF8	0.665	0.053	12.620	< 0.001	0.562	0.768	
	PF9	0.531	0.057	9.366	< 0.001	0.420	0.642	
	PF10	0.977	0.050	19.634	< 0.001	0.879	1.074	
	PF11	0.949	0.053	17.895	< 0.001	0.845	1.053	
	PF12	1.066	0.045	23.898	< 0.001	0.979	1.154	
	PF13	1.039	0.047	22.169	< 0.001	0.947	1.131	
Physical Education Course Interest	PECI1	0.999	0.043	23.081	< 0.001	0.914	1.084	
	PECI2	1.013	0.042	24.169	< 0.001	0.931	1.095	
	PECI3	1.042	0.042	24.899	< 0.001	0.960	1.124	
	PECI4	0.931	0.040	23.245	< 0.001	0.853	1.010	
	PECI5	1.076	0.041	26.054	< 0.001	0.995	1.156	0.952
	PECI6	0.953	0.043	22.317	< 0.001	0.869	1.037	
	PECI7	0.794	0.036	22.210	< 0.001	0.724	0.864	
	PECI8	0.859	0.042	20.499	< 0.001	0.777	0.941	
	PECI9	0.829	0.038	22.049	< 0.001	0.755	0.902	
	PECI10	0.961	0.040	23.943	< 0.001	0.882	1.040	
School Attachment	SA1	1.131	0.042	27.085	< 0.001	1.049	1.213	
	SA2	1.171	0.039	29.894	< 0.001	1.094	1.248	0.900
	SA3	0.986	0.043	23.114	< 0.001	0.902	1.069	
	SA4	0.960	0.053	18.010	< 0.001	0.855	1.064	

The correlations of the variables with each other, as well as their mean, standard deviation (SD) values, skewness, and kurtosis values, were presented. The mean values and standard deviations of the variables were found as follows, respectively: PAS (M = 5.016, SD = 1.626), PF (M = 3.338, SD = 0.884), PECI (M = 4.181, SD = 0.973), and SA (M = 4.071, SD = 1.122). Skewness and kurtosis values were calculated as −0.964 and 0.205 for PAS, −0.527 and 0.006 for PF, −1.563 and 1.849 for PECI, and −1.247 and 0.609 for SA. These values show that the distribution of the variables is close to a normal distribution. When the correlation values between perceived autonomy support (PAS), perceived feedback from the physical education teacher (PF), physical education course interest (PECI), and school attachment (SA) are analyzed, it is seen that there is a positive and significant correlation between PAS and PF (*r* = 0.697, *p* < 0.001). There were also significant positive correlations between PAS and PECI (*r* = 0.663, *p* < 0.001) and SA (*r* = 0.511, *p* < 0.001). Significant positive correlations were also observed between PF and PECI (*r* = 0.682, *p* < 0.001) and SA (*r* = 0.506, *p* < 0.001). Finally, there was a positive correlation between PF and SA (*r* = 0.571, *p* < 0.001) According to Hopkins et al. (2009), correlation coefficients can be interpreted as follows: trivial (*r* ≤ 0.1), small (*r* = 0.1–0.3), moderate (*r* = 0.3–0.5), large (*r* = 0.5–0.7), very large (*r* = 0.7–0.9), and almost perfect (*r* ≥ 0.9). Based on this classification, the correlations between PAS and PF (*r* = 0.697), PF and PECI (*r* = 0.682), and PAS and PECI (*r* = 0.663) are considered large. The correlations between PAS and SA (*r* = 0.511), PF and SA (*r* = 0.506), and PECI and SA (*r* = 0.571) are also in the moderate to large range (Table 2).

**Table 2:** Mean, standard deviation, skewness, and kurtosis values of perceived autonomy support, perceived feedback, physical education course interest, and school attachment.

Variables	PAS	PF	PECI	SA	Mean	SD	Skewness	Kurtosis
PAS	-				5.016	1.626	−0.964	0.205
PF	0.697***	-			3.338	0.884	−0.527	0.006
PECI	0.663***	0.682***	-		4.181	0.973	−1.563	1.849
SA	0.511***	0,506***	0.571***	-	4.071	1.122	−1.247	0.609

PAS, Perceived Autonomy Support; PF, Perceived Feedback; PECI, Physical Education Course Interest; SA, School Attachment. *p < 0.05, **p < 0.01, ***p < 0.001.

In this study, the effects of perceived feedback from the physical education teacher and perceived autonomy support variables on school attachment were examined. The results show that the direct effect of perceived feedback from the physical education teacher on school attachment (B = 0.154, z = 2.644, *p* = 0.008, 95% CI [0.04, 0.268]) was moderate in size, and the direct effect of perceived autonomy support on school attachment (B = 0.109, z = 3.527, *p* < 0.001, 95% CI [0.048, 0.17]) was small. It was found that perceived feedback from the physical education teacher (B = 0.485, z = 10.643, *p* < 0.001) had a very large effect on physical education course interest (R^2^ = 0.534; f^2^ = 1.146), and perceived autonomy support (B = 0.224, z = 9.066, *p* < 0.001) had a large effect. Physical education course interest had a positive and significant effect on school attachment (B = 0.361, z = 7.330, *p* < 0.001), which was classified as a large effect (R^2^ = 0.366; f^2^ = 0.577). In addition, it was detected that the indirect effect of perceived feedback from the physical education teacher on school attachment through physical education course interest (B = 0.175, z = 6.037, *p* < 0.001, 95% CI [0.118, 0.232]) was moderate, and the indirect effect of perceived autonomy support on school attachment through physical education course interest (B = 0.081, z = 5.7, *p* < 0.001, 95% CI [0.053, 0.109]) was small. When the total effects were analyzed, the total effect of perceived feedback from the physical education teacher on school attachment (B = 0.329, z = 5.918, *p* < 0.001, 95% CI [0.22, 0.438]) was large, and the total effect of perceived autonomy support on school attachment (B = 0.190, z = 6.290, *p* < 0.001, 95% CI [0.131, 0.249]) was moderate. Effect size classifications were made based on Cohen (1988) thresholds for f^2^: 0.02 (small), 0.15 (medium), and 0.35 (large). These findings reveal essential factors influencing school attachment both directly and indirectly (Table 3).

**Table 3:** Direct, indirect, and total effects of the model created according to the hypotheses.

Effect type	Predictor → Outcome	Estimate	Std. Error	z-value	*p*-value	95% CI lower	95% CI upper
Direct Effects	PF → SA	0.154	0.058	2.644	0.008	0.04	0.268
	PAS → SA	0.109	0.031	3.527	< 0.001	0.048	0.17
	PECI → SA	0.361	0.049	7.330	< 0.001	0.265	0.458
	PF → PECI	0.485	0.046	10.643	< 0.001	0.395	0.574
	PAS → PECI	0.224	0.025	9.066	< 0.001	0.176	0.273
Indirect Effects	PF → PECI → SA	0.175	0.029	6.037	< 0.001	0.118	0.232
	PAS → PECI → SA	0.081	0.014	5.7	< 0.001	0.053	0.109
Total Effects	PF → SA	0.329	0.056	5.918	< 0.001	0.22	0.438
	PAS → SA	0.190	0.030	6.290	< 0.001	0.131	0.249

PAS, Perceived Autonomy Support; PF, Perceived Feedback; PECI, Physical Education Course Interest; SA, School Attachment.

**Figure 1:**
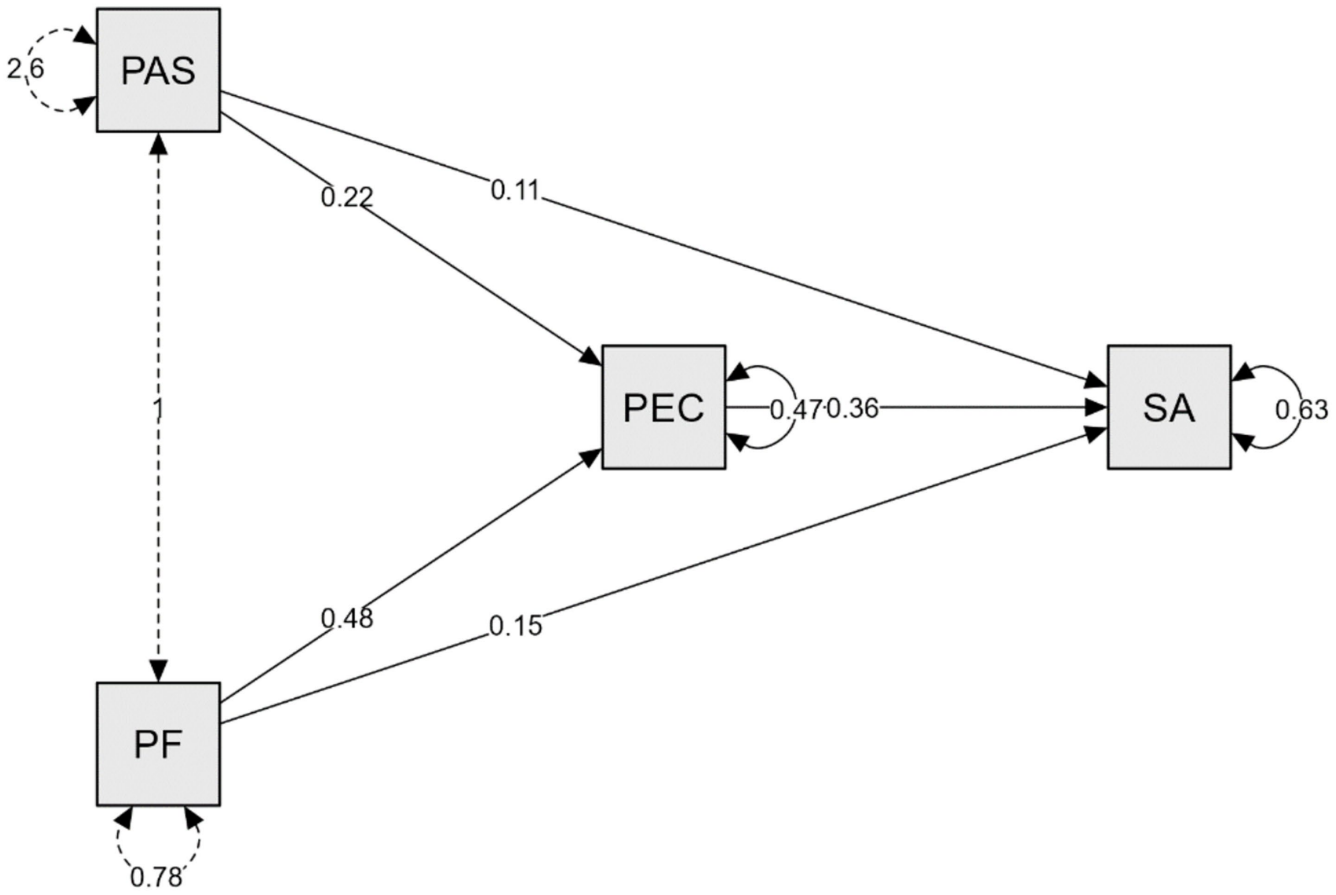
Path analysis.

Figure 1 shows the statistical results for the direct, indirect and mediating effects between the variables.

## 6 Discussion

In this study, it was examined how the autonomy support and feedback provided by teachers in PE (physical education) courses affect students’ school attachment through their interest in PE classes. The results of our research showed that autonomy support and feedback significantly affected physical education course interest and school attachment. Moreover, the mediating role of PE course interest in these relationships was confirmed. According to the research results, autonomy support positively affected PE course interest (H1). Research shows that when students feel their PE teachers support their autonomy, it positively affects their intrinsic motivation, engagement, and overall interest in PE lessons (Ommundsen and Kvalø, 2007; Ulstad et al., 2018). This finding aligns with Deci and Ryan (2000) self-determination theory and suggests that autonomy support strengthens students’ motivation by giving them more control over their learning process. Zhang et al. (2012) found that teachers’ autonomy support positively affected students’ expectations, task values, and concentration, while similarly, Reeve et al. (2004) reported that autonomy-supportive instructional strategies increased students’ interest and academic performance. These results suggest that autonomy support increases student motivation and supports PE course interest and overall academic achievement. It is understood that teachers giving students more freedom in their learning processes plays a role in increasing students’ interest in the course.

Second, it was confirmed that autonomy support directly and positively affected school attachment (H2). Perceived autonomy support from PE teachers has been consistently associated with various positive outcomes for students. Studies have shown that when teachers exhibit autonomy-supportive behaviors in PE classes, such as respecting students’ attitudes, offering choices, and showing patience, students tend to experience higher levels of intrinsic motivation (Chang et al., 2016; Leptokaridou et al., 2016). This autonomy support has been found to predict school attachment through a sequence of motivation that includes students’ perceived effort and physical self-esteem (Standage et al., 2005). Autonomy support creates a supportive and empowering environment that encourages students to take responsibility for learning and decision-making. This kind of support leads students to feel more competent and engaged in the school context (Fin et al., 2019). While it is emphasized that autonomy support strengthens their school attachment by increasing students’ motivation in other research, it is observed that intrinsically motivated students show more interest in learning and school activities and develop positive attitudes toward school. This enables them to establish stronger emotional bonds with their educational environment.

Thirdly, it was found that PE course interest played a mediating role in the relationship between autonomy support and school attachment. This result supports hypothesis H3, which suggests that PE course interest mediates the relationship between autonomy support and school attachment. This finding is consistent with previous studies (Cheon et al., 2012; Haerens et al., 2015) and demonstrates how students’ interest in courses positively affects their overall school attachment. Students’ interest in the PE course increases thanks to the autonomy support they receive from their teachers, and this interest reinforces their commitment to the school. In their study, Vallerand et al. (1997) reported that autonomy support increased students’ interest in the course, and this interest reinforced their school attachment. Van den Berghe et al. (2014) study reveals that autonomy-supportive teaching strategies increase students’ interest in the PE course, leading them to participate more in the lessons, and this interest reinforces students’ overall school attachment. This study emphasizes that teachers’ autonomy-supportive approaches play a role in increasing students’ motivation and interest in lessons, which in turn have positive effects on school attachment.

Fourth, it was detected that perceived feedback from the PE teacher had a positive and significant effect on students’ interest in the PE course (H4). It is understood that students’ interest in the lessons is reinforced through the feedback provided by their teachers. Research shows that constructive and supportive feedback provided by teachers significantly increases students’ interest in physical education courses (Koka and Hein, 2006; Mouratidis et al., 2008). The effect of feedback on student interest becomes even more evident with the constructive and supportive feedback that teachers provide. Especially in PE courses, which are practice-oriented, positive feedback from teachers helps students participate more actively in the lessons (Chen, 2001; Morgan and Hansen, 2008). However, demographic variables such as gender, academic level, and learning styles can affect the effects of feedback. For example, in their study, Harks et al. (2013) concluded that students with low academic achievement need more feedback and that it is more effective in increasing their motivation and interest in the lessons. On the other hand, it is reported that students with high academic achievement find feedback less motivating, and therefore, their interest in the lessons is less affected by feedback (Kluger and DeNisi, 1996). Consequently, it is vital for teachers to consider these demographic differences when using feedback strategies.

Fifth, the findings of our study demonstrate that feedback from PE teachers has a positive and significant effect on students’ school attachment (H5). This finding is crucial for understanding how student-teacher interactions and feedback shape students’ engagement in their school life. In particular, the quality of feedback and how students perceive it can be decisive factors in school attachment. The positive feedback that students receive from their teachers contributes to students feeling more valued and successful, which in turn leads them to feel more connected to school (Hattie and Timperley, 2007). While the positive effects of feedback on students’ motivation and academic achievement have been widely documented, the effects of feedback on students’ school attachment have been less studied. One of the critical findings of this study is how feedback, especially in physical education courses, plays a role in increasing students’ school attachment.

Sixth, our research findings indicate that PE course interest has a mediating role in the effect of feedback from PE teachers on school attachment (H6). This finding indicates that teacher feedback not only provides an immediate motivational boost but also strengthens students’ overall school attachment by increasing their interest in PE courses in the long run. Studies on how constructive feedback provided by teachers in PE lessons increases students’ interest in the lesson and, thus, their attachment to school show that these courses contribute to students’ physical and social development (Koka, 2013). The study by Mouratidis et al. (2008) reveals that teachers’ constructive feedback reinforces students’ overall school engagement by increasing their interest and motivation in the courses. In this context, it is observed that PE course interest plays a crucial mediating role in the relationship between teacher feedback and school attachment.

The results obtained in this study demonstrate that the proposed model is statistically significant and theoretically consistent. However, due to the use of cross-sectional data, a definitive one-way causation cannot be established. For example, it is also possible that students are more interested in PE courses because they have higher levels of school attachment (reverse causality possibility). Furthermore, the fact that all data were collected over the same period and using self-reporting methods may increase the effect of shared method variance. In future studies, these effects can be controlled by using different data collection tools (Podsakoff et al., 2003).

## 7 Conclusion

In this study, how autonomy support and feedback provided by teachers in physical education classes affect students’ engagement with the school through their interest in physical education classes was examined. The findings of the study demonstrated that autonomy support and feedback significantly affected students’ interest in physical education courses and school attachment. Furthermore, the mediating role of physical education course interest in these relationships was confirmed. It was observed that when students perceived autonomy support and feedback positively, their interest in physical education courses increased, and this increase reinforced their school attachment. These results suggest that teachers’ autonomy-supportive and constructive feedback strategies increase students’ interest in courses and overall school attachment.

## 8 Limitations and suggestions for future research

This study has several limitations. First, the study data were collected in a cross-sectional design, which makes it difficult to identify causal relationships. For future studies, longitudinal designs could be used to examine the change and impact of these relationships over time in more depth. Second, this study is based only on students’ perceptions. The study was conducted only in schools in a specific region; therefore, the generalizability of the findings is limited. Further studies in different regions and cultural contexts will increase the generalizability of the results. Studies evaluating teachers’ feedback-giving behaviors and autonomy-supportive attitudes with objective criteria can increase the validity of the findings. In addition, examining the effects of feedback in terms of demographic variables such as gender, academic level, and learning styles may reveal the effects of these factors on feedback strategies in more detail.

Moreover, this study did not consider potential moderating factors that may influence the effects of perceived feedback and autonomy support, such as students’ age, socio-economic status, future academic expectations, or participation in extracurricular sports activities. Future research could explore how these demographic and contextual variables interact with teacher behaviors to better understand the differential impact of feedback strategies on student motivation and school attachment.

## 9 Implications

The findings of this study provide valuable insights for educators and teacher education programs. PE teachers can increase students’ interest in lessons and strengthen school attachment by offering constructive feedback and autonomy-supportive practices. These strategies foster students’ motivation, leading to higher engagement in lessons and a stronger emotional connection to the school. Therefore, teacher training and professional development programs should include modules focused on autonomy support and feedback strategies. These results, grounded in Self-Determination Theory (Deci and Ryan, 2000), suggest several concrete classroom strategies. Autonomy support can be implemented through behaviors such as offering meaningful choices, acknowledging students’ perspectives, encouraging goal setting, and reducing controlling language (Reeve, 2006). For feedback, its effectiveness increases when it is specific, timely, and focused on effort or strategies rather than outcomes alone (Hattie and Timperley, 2007). In PE settings, this might involve recognizing individual progress, encouraging peer collaboration, and setting personalized goals. Such practices not only satisfy students’ psychological needs but also translate into increased interest in lessons and long-term school attachment.

## Data Availability

The raw data supporting the conclusions of this article will be made available by the authors, without undue reservation.
